# Exosomes from antler stem cells alleviate mesenchymal stem cell senescence and osteoarthritis

**DOI:** 10.1007/s13238-021-00860-9

**Published:** 2021-08-03

**Authors:** Jinghui Lei, Xiaoyu Jiang, Wei Li, Jie Ren, Datao Wang, Zhejun Ji, Zeming Wu, Fang Cheng, Yusheng Cai, Zheng-Rong Yu, Juan Carlos Izpisua Belmonte, Chunyi Li, Guang-Hui Liu, Weiqi Zhang, Jing Qu, Si Wang

**Affiliations:** 1 Advanced Innovation Center for Human Brain Protection, National Clinical Research Center for Geriatric Disorders, Xuanwu Hospital Capital Medical University, Beijing, 100053 China; 2 State Key Laboratory of Membrane Biology, Institute of Zoology, Chinese Academy of Sciences, Beijing, 100101 China; 3 State Key Laboratory of Stem Cell and Reproductive Biology, Institute of Zoology, Chinese Academy of Sciences, Beijing, 100101 China; 4 Aging Translational Medicine Center, Xuanwu Hospital, Capital Medical University, Beijing, 100053 China; 5 CAS Key Laboratory of Genomic and Precision Medicine, Beijing Institute of Genomics, Chinese Academy of Sciences, Beijing, 100101 China; 6 Institute for Stem Cell and Regeneration, Chinese Academy of Sciences, Beijing, 100101 China; 7 Institute of Antler Science and Product Technology, Changchun Sci-Tech University, Changchun, 130000 China; 8 University of Chinese Academy of Sciences, Beijing, 100049 China; 9 National Laboratory of Biomacromolecules, CAS Center for Excellence in Biomacromolecules, Institute of Biophysics, Chinese Academy of Sciences, Beijing, 100101 China; 10 China National Center for Bioinformation, Beijing, 100101 China; 11 Beijing Institute for Stem Cell and Regenerative Medicine, Beijing, 100101 China; 12 Department of Orthopaedics, Peking University First Hospital, Beijing, 100034 China; 13 Gene Expression Laboratory, Salk Institute for Biological Studies, La Jolla, CA USA

**Dear Editor,**

Stem cell therapy holds enormous and revolutionary promise to treat various age-related diseases, such as diabetes, heart failure, and Parkinson’s disease. However, low retention and survival rate of delivered stem cells, partially due to immunological rejection, constitute major hurdles for the clinical implementation of stem cell therapy (Lei et al., 2021a). Since mounting evidence showed that several types of stem cells mainly exert their therapeutic effects through the secretion of paracrine effects, exosomes, which are released by stem cells and execute most paracrine functions, have begun to draw attention in the field (Tran and Damaser, 2015). Exosomes are membrane-enclosed vesicles with an average diameter of ~100 nanometers secreted by the cells, containing cytokines, growth factors, signaling lipids, mRNAs, and regulatory miRNAs for intercellular communications. Compared to transplanted stem cells, exosomes are safer to apply due to their cell-free properties. Therefore, exosome-based treatment could serve as a promising therapeutic strategy for human diseases. However, exosomes are highly heterogeneous in their sizes, contents and activities, mostly reflective of their various cell types of origin (Kalluri and LeBleu, 2020). These differences may subsequently influence the therapeutic efficacy of exosomes. Therefore, a cell source that can be constantly scaled up for producing exosomes of large quantity and with high potency would greatly facilitate the development of exosome-based therapeutic strategies.

So far, velvet antler is the only known mammalian organ that can regenerate as a whole annually. Its regeneration is initiated by antler stem cells (ASCs), which are capable of sustained self-renewal and differentiation into multi-lineage cells to form an organ (Sui et al., 2020). Compared with other types of mammalian stem cells, ASCs are much easier to acquire and exhibit much higher proliferative and regenerative capacity (Wang et al., 2019; Rong et al., 2020). As a result, ASCs could be a potentially inexhaustible source to support exosome-based treatment. In this study, we set out to investigate the possible effects of ASC-derived exosomes on the rejuvenation of senescent human stem cells and the alleviation of the symptoms of osteoarthritis (OA) (Fig. 1A).

**Figure 1:**
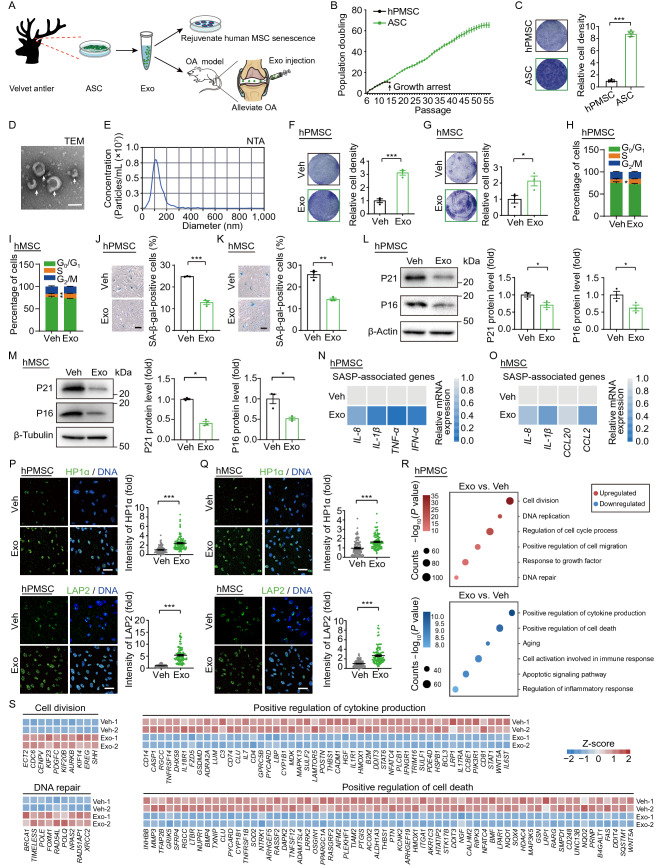
**Exosomes derived from antler stem cells (ASCs) alleviate senescence in human MSCs** ***in vitro***. (A) Schematic diagram of the purification of exosomes from ASC-conditioned medium, which were then supplemented for culture of senescent human MSCs (including human primary MSCs and hESC-derived hMSCs) or used for intra-articular injection in the OA mouse model. ASC, antler stem cell; Exo, exosome; OA, osteoarthritis. (B) Growth curve showing that ASCs have a much higher self-renewal capacity than hPMSCs. Data are presented as the mean ± SEMs, *n* = 3. (C) Representative images and statistical analysis of the clonal expansion ability of ASCs and relative to that of hPMSCs at passage 12. Data are shown as the mean ± SEMs, *n* = 6. ***, *P* < 0.001 (two-tailed *t* test). (D) Morphology of exosomes under transmission electron microscopy (TEM). Scale bar, 100 nm. (E) Particle size distribution of exosomes measured by nanoparticle tracking analysis (NTA). (F and G) Clonal expansion analysis of hPMSCs (F) or hMSCs (G) treated by vehicle (Veh) or exosomes (Exo). The relative cell density is quantified as fold changes (Exo vs. Veh) and presented as the mean ± SEMs, *n* = 3. *, *P* < 0.05; ***, *P* < 0.001 (two-tailed *t* test). (H and I) Cell cycle analysis of hPMSCs (H) or hMSCs (I) with Veh or Exo treatment. Data are shown as the mean ± SEMs, *n* = 3. *, *P* < 0.05; **, *P* < 0.01 (two-tailed *t* test). (J and K) SA-β-gal staining of hPMSCs (J) or hMSCs (K) treated by Veh or Exo. Scale bars, 50 μm. The percentages of  SA-β-gal-positive cells are quantified and presented as the mean ± SEMs, *n* = 3. **, *P* < 0.01; ***, *P* < 0.001 (two-tailed *t* test). (L and M) Representative western blot images and quantifications of P16 and P21 expression in exosome-treated (Exo) relative to vehicle-treated (Veh) hPMSCs (L) or hMSCs (M). Data are shown as the mean ± SEMs, *n* = 3. *, *P* < 0.05 (two-tailed *t* test). (N and O) Heatmaps showing the relative mRNA expression levels of the SASP-associated genes in Veh or Exo-treated hPMSCs (N) or hMSCs (O). The average expression levels of the genes in Exo treated hPMSCs (N) or hMSCs (O) were normalized to those cells treated with Veh. *n* = 3. (P and Q) Representative immunofluorescence images of HP1α (upper) or LAP2 (lower) in Veh or Exo treated hPMSCs (P) or hMSCs (Q). Relative fluorescence intensity of HP1α or LAP2 was quantified. Scale bars, 50 μm. 100 cells were assessed in each group. ***, *P* < 0.001 (two-tailed *t* test). (R) Dot plots showing significantly enriched terms of upregulated or downregulated genes in hPMSCs treated by Veh or Exo. The size of each circle represents the number of genes enriched for each term. (S) Heatmaps showing the relative expression levels of differently expressed genes associated with indicated terms in hPMSCs treated with Exo vs. Veh

First, we determined the *in vitro* self-renewal capacity of ASCs. Compared to the widely used human primary mesenchymal stem cells (hPMSCs), ASCs demonstrated a much higher proliferation capacity represented by growth curve recording and clonal expansion assay (Fig. 1B and 1C), suggesting ASCs as a superior, stable, and sustainable stem cell resource for exosomes. Next, we examined the basic features of exosomes collected from the conditioned medium of ASCs. Transmission electron microscopy (TEM) and nanoparticle tracking analysis (NTA) showed that the collected exosomes displayed a classical cup- or sphere-shaped morphology with an average diameter of ~100 nanometers (Fig. 1D and 1E). Since a prior report showed that ASCs conferred antler development and regeneration function primarily via their secretory factors and cytokine contents (Sui et al., 2020), we analyzed the protein components of ASC-derived exosomes via LC-MS/MS assay (Fig. S1A). In total, 438 proteins were identified and listed in Table S1. Core pathways determined by Gene Ontology (GO) enrichment analysis of these proteins revealed several regeneration-related pathways, such as “response to growth factor” and “blood vessel development” (Fig. S1B). To further dissect the potential contributing factors of ASC-derived exosomes in regeneration, we ranked the top 10 proteins based on their score value. Of note, 7 out of the 10 top proteins have been found to perform major functions in promoting cell proliferation and migration, negative regulation of apoptosis, or inhibiting inflammation (Fig. S1C). Overall, our observations implied a positive effect of ASC-derived exosomes on stem cell regeneration.

Next, we investigated whether ASC-derived exosomes could attenuate human stem cell senescence. Upon exosome treatment, the senescent phenotypes of hPMSCs and human ESC-derived MSCs (hMSCs) at late passages were substantially mitigated, as shown by improved clonal expansion ability, elevated percentage of cells in S phase (synthesis phase), and decreased senescence-associated-β-galactosidase (SA-β-gal) activity (Fig. 1F–K). Additionally, typical indicators of cellular senescence including protein levels of P16 and P21, as well as the expression levels of senescence-associated secretory phenotype (SASP)-related genes (*IL-8*, *IL-1β*, etc.), were reduced in exosome-treated hPMSCs or hMSCs compared to controls (Exo vs. Veh) (Fig. 1L–O). Moreover, diminished levels of heterochromatin-associated proteins HP1α and nuclear lamina proteins LAP2, a well-known driving force for hMSC senescence (Bi et al., 2020), were partially rescued by exosome treatment (Fig. 1P and 1Q). In order to reveal the underlying mechanism of the geroprotective effects of ASC-derived exosomes, we compared the transcriptomes of aged hPMSCs subjected to exosome and vehicle treatment. In accordance with the retarded senescent phenotypes, we observed upregulation of genes involved in “cell division” (e.g., *ECT2*, *CDC6*, *CENPV*), “response to growth factor” (e.g., *FOS*, *JUN*) and “DNA repair” (e.g., *BRCA1*, *TIMELESS*, *POLE*) in exosome-treated hPMSCs. By comparison, genes related to “positive regulation of cytokine production” (e.g., *CD14*, *CASP1*, *RGCC*), “positive regulation of cell death” (e.g., *INHBB*, *MMP3*, *TFAP2B*) and “aging” (e.g., *ID2*, *MB21D1*) were downregulated in hPMSCs treated with ASC-derived exosomes (Figs. 1R, 1S, S1D and S1E, and Table S2). Collectively, these data indicated that the treatment of ASC-derived exosomes attenuated senescent phenotypes in human mesenchymal stem cells, probably by promoting self-renewal and repressing the senescence-related inflammatory responses.

Development and progression of OA have been reported to be induced by the accumulation of senescent cells in or near joints (Xu et al., 2017), thus attenuation of cellular senescence in these cells may present an option for OA treatment. As mentioned before, ASC-derived exosomes could rejuvenate senescent human MSCs. In addition, ASCs possess osteogenic and chondrogenic capabilities, as they contribute to the annual regeneration of bone and cartilage in the antler (Wang et al., 2019). We therefore, wondered whether treatment with ASC-derived exosomes could ameliorate OA characterized by accumulation of senescent cells and attrition of protective cartilage. The OA mouse model was induced by the anterior cruciate ligament transection (ACLT) surgery, followed by intra-articular exosome or vehicle administration as illustrated in the workflow (Fig. 2A). Through the grip strength test, we observed a decreased level of grip strength in ACLT-induced OA mice (Fig. 2B). Notably, this was partially restored by intra-articular injections of ASC-derived exosomes (Fig. 2B). In addition, microcomputed tomography (micro-CT) analysis revealed an apparent bone erosion in ACLT-induced OA mice and a marked amelioration with exosome treatment (Fig. 2C). A subsequent histological assessment showed that cartilage degeneration after ACLT surgery was rescued in the exosome-treated group (Fig. 2D). Furthermore, an increased expression of the cellular proliferation marker Ki67 and a decreased expression of the senescence marker P16 were detected in the articular cartilage zone of exosome-treated mice (Fig. 2E). Moreover, RNA-seq analysis of the mouse joints revealed a set of OA-associated differentially expressed genes (DEGs) whose expression patterns were reversed upon intra-articular administration of ASC-derived exosomes (Figs. 2F and S1F). Among the downregulated OA-associated DEGs reversed by exosomes (hereafter referred as to “Down-DEGs rescued by Exo”) were genes enriched in “tissue morphogenesis”, “stem cell differentiation”, and “multicellular organism growth” (Fig. 2G, 2H and Table S2). In contrast, upregulated OA-associated DEGs reversed by exosomes (hereafter referred as to “Up-DEGs rescued by Exo”) were involved in “apoptotic signaling pathway”, “response to reactive oxygen species” and “regulation of immune effector process” (Fig. 2G, 2H and Table S2). To confirm the RNA-seq results, we tested and verified some of the affected genes involved in “tissue morphogenesis” and “stem cell differentiation” (*Foxc1*), “neutrophil mediated immunity” and “apoptotic process” (*Csf3r*, *Lcn2*, *Tnfrsf1a*, *Atp2a1*) by RT-qPCR (Fig. 2I). To further evaluate the common molecular mechanism underlying alleviation of cellular senescence and OA by exosome treatment, we compared the DEGs in hPMSCs and reversed DEGs in the joint tissues of mice with OA upon treatment. We found 25 upregulated and 5 downregulated genes shared by these two groups (Fig. S1G). However, when we analyzed the upstream regulators of the aforementioned overlapping genes by ingenuity pathway analysis (IPA), we observed 151 and 420 upstream regulators of the upregulated or downregulated genes shared by hPMSC and OA mouse models, respectively (Fig. 2J), suggesting conserved upstream regulatory pathways targeted by exosomes. Of note, the upstream regulators of upregulated genes were mainly classified as growth factors (e.g., FGF2, AGT, NGF) or transcription factors to promote self-renewal (e.g., ZNF217, FOS, SOX2) (Fig. 2K and Table S3). By contrast, the upstream regulators of downregulated genes were enriched for inflammatory factors (e.g., TNF, IL1B, IFNG) or transcription factor antagonizing proliferation (e.g., TP53) (Fig. 2K and Table S3). Taken together, our data indicated that intra-articular administration of ASC-derived exosomes contributed to bone and cartilage regeneration and thus rectified the symptoms of OA, probably resulted from the attenuation of senescence and inflammatory responses in OA (Fig. 2L).

**Figure 2:**
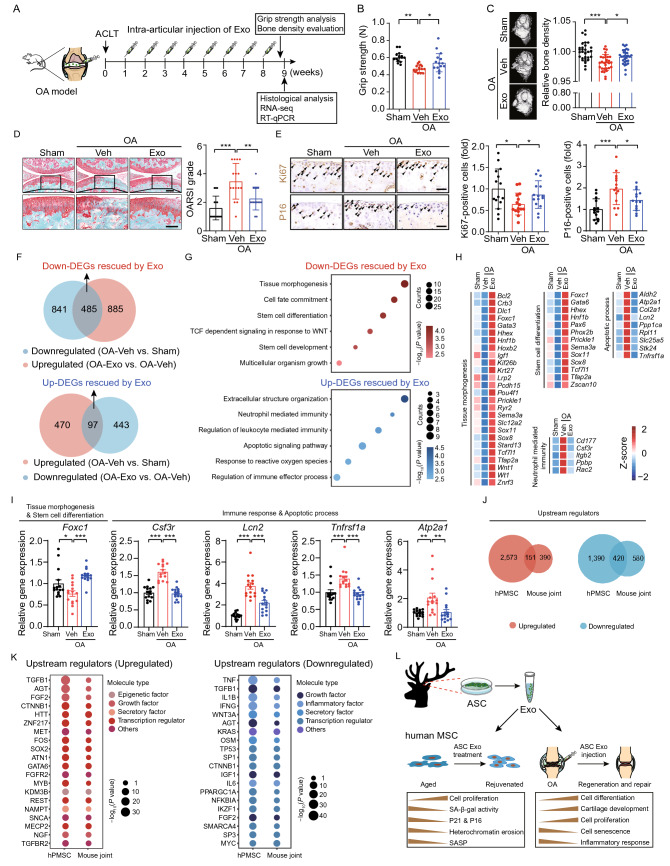
**Intra-articular injection of exosomes derived from antler stem cells alleviates ACLT-induced OA in mice**. (A) Schematic diagram of the experimental procedures. ACLT, anterior cruciate ligament transection. (B) Grip strength test in the OA mouse model with Veh or Exo treatment. Data are shown as the mean ± SEMs, *n* = 15 mice. *, *P* < 0.05, **, *P* < 0.01 (one-way ANOVA followed by Dunnett’s test). (C) Bone density analysis of OA mouse joints with Veh or Exo treatment. Data are shown as the mean ± SEMs, *n* = 15 mice (Both hindlimb joints were tested for each mouse). *, *P* < 0.05, ***, *P* < 0.001 (one-way ANOVA followed by Dunnett’s test). (D) Representative images of safranin O and fast green staining and quantification of Osteoarthritis Research Society International (OARSI) grade of articular cartilages. Scale bars, 200 μm. Data are shown as the mean ± SEMs, *n* = 15 mice. **, *P* < 0.01, ***, *P* < 0.001 (one-way ANOVA followed by Dunnett’s test). (E) Immunohistochemical staining for Ki67 (upper) and P16 (lower) and quantitative analysis in articular cartilage from OA mice treated by Veh or Exo. Scale bars, 60 μm. Data are shown as the mean ± SEMs, *n* = 15 mice. *, *P* < 0.05, ***, *P* < 0.001 (one-way ANOVA followed by Dunnett’s test). (F) Venn diagrams showing the number of downregulated (upper) or upregulated (lower) OA-associated DEGs and the number of rescued DEGs in the joint tissues of mice with OA upon Exo treatment. The number of indicated overlapping genes was also shown. (G) Enrichment analysis of downregulated (upper) or upregulated (lower) OA-associated DEGs rescued by Exo in mouse joint. (H) Heatmaps showing the relative expression levels of DEGs associated with indicated terms and pathways in the joints of sham mice or OA mice treated with Veh or Exo. (I) RT-qPCR analysis verified the changes of indicated genes involved in the indicated pathways. *n* = 15 mice. Data are shown as the mean ± SEMs, *, *P* < 0.05, **, *P* < 0.01, ***, *P* < 0.001 (one-way ANOVA followed by Dunnett’s test). (J) Venn diagrams showing the number of overlapping upstream regulators of DEGs in hPMSCs upon Exo treatment and rescued DEGs in the joints of OA mice after intra-articular administration of Exo based on the Ingenuity Pathway Analysis (IPA). (K) Dot plots showing the top 20 upstream regulators shared by upregulated or downregulated genes in Exo-treated hPMSCs and joint tissues of Exo-treated OA mice. The top 20 upstream regulators were listed based on their *P* value. (L) A schematic illustration showing the beneficial effects of ASC-derived exosomes in rejuvenating human cellular senescence *in vitro* and alleviating OA *in vivo*

To summarize our observations, administration of exosomes secreted from ASCs alleviated human stem cell senescence, and attenuated cartilage degeneration in the OA mouse model as well. Mechanistically, ASC-derived exosomes exert their geroprotective effects by promoting cell division and repressing the senescence-associated inflammation both *in vitro* and *in vivo*. Collectively, our study suggests ASC-derived exosomes as a potential biomaterial to develop cell-free therapy against aging and age-related diseases.

In this study, we found that ASCs exhibit improved self-renewal ability compared with hPMSCs. Consistent with our findings, ASCs were reported to have higher self-renewal capacity and *ex-vivo* expansion ability in comparison with other types of MSCs (Wang et al., 2019). Additionally, as ASCs were observed to possess osteogenic and chondrogenic differentiation potentials, we hypothesized that exosomes secreted by ASCs may exert regeneration-promoting functions, thus facilitating cartilage repair and remodeling. Indeed, we found that ASC-derived exosomes can alleviate OA through defying senescence of the intra-articular cells. Consistently, previous studies demonstrated that the antler extract has multiple biological activities including anti-inflammation and wound healing (Yao et al., 2018). Moreover, ASCs and ASC-conditioned medium have been tested and confirmed for their beneficial effects in some animal disease models (Rong et al., 2019; Rong et al., 2020).

Our proteomics data analysis revealed that ASC-derived exosomes were packed with protein effectors in response to growth factors, probably contributing to their rejuvenating effects on aged human mesenchymal stem cells and in mouse OA models. In line with our results, a recent study showed that extracellular vesicles (EVs) secreted by neonatal umbilical cord-derived MSCs can revive senescent adult bone marrow-derived MSCs via the transfer and deposition of the mRNA transcript of proliferating cell nuclear antigen (*PCNA*) (Lei et al., 2021b). These observations suggest that the youthful mesodermal progenitor-derived exosomes contain abundant geroprotective factors. Therefore, it is critical and worthwhile to uncover the effective molecules in exosomes responsible for delaying aging and alleviating age-related diseases.

Our data, for the first time, showed that ASC-derived exosome-based treatment can stabilize heterochromatin and revitalize aged human MSC. Consistently, we discovered that loss of heterochromatin is a driving force for cellular senescence, and geroprotective compounds like Vitamin C and quercetin can delay the accelerated cellular senescence at least in part via facilitating the maintenance of heterochromatin organization in the previous studies (Geng et al., 2019; Bi et al., 2020). We have also identified a panel of heterochromatin stabilizers including DGCR8, CLOCK and CBX4 that can revive senescent human MSC, thus lentiviral-mediated overexpression and delivery of these “rejuvenation” factors can mitigate symptoms of age-related OA (Zhang et al., 2020). Previously, local clearance of senescent cells by senolytic molecules was reported to attenuate the development of post-traumatic OA (Jeon et al., 2017). Therefore, administration of ASC-derived exosomes, in combination with transduction of heterochromatin stabilizers, may represent a novel therapeutic strategy against different types of OA through revivifying the senescent cells. Of note, exosomes have been proven to possess superior characteristics of safety and lower immunogenicity in treating various human diseases (Yin et al., 2019). ASC-derived exosomes, although a new comer, hold a potential as biomaterial for treatment of OA and other age-related disorders in the future.

As in the development of any novel treatment, there are a number of concerns to be addressed before advancing the current findings towards a clinical application. For one thing, the effects of ASC-derived exosomes in physiologically aged OA via mouse model and other large animal models require further evaluation. Moreover, a comprehensive study including longer-term safety monitoring is needed to exclude pro-tumorigenic effects. Additional safety issues in treating human diseases with biomaterial from a different species, (although not very likely but) may involve elicitation of the immunological rejection, cytokine release syndrome, infusional toxicities, etc., all of which await in-depth investigation in the future. In summary, ASC-derived exosomes hold a potential in being used as a novel geroprotective strategy for ameliorating stem cell senescence and treating OA, paving the way for development of new treatments against aging-related diseases.

## Supplementary Information

Below is the link to the electronic supplementary material.

Supplementary material 1 (PDF 556 kb)

Supplementary material 2 (XLS 151 kb)

Supplementary material 3 (XLS 740 kb)

Supplementary material 4 (XLS 1400 kb)

Supplementary material 5 (XLS 42 kb)

Supplementary material 6 (XLSX 11 kb)
