# Supplementary material for: Exosomes from antler stem cells alleviate mesenchymal stem cell senescence and osteoarthritis
Source: Protein Cell. 2021 Aug 3;13(3):220–6. doi: 10.1007/s13238-021-00860-9 (PMC8901817; doi:10.1007/s13238-021-00860-9)

MATERIALS AND METHODS

Generation of hMSCs and hPMSCs

hESC-derived MSCs (hMSCs) were generated from hESCs following previous study (Liang et al., 2021). Briefly, hESCs were digested to obtain embryoid bodies (EBs) in differentiation medium (DMEM-F12 medium (GIBCO) supplemented with 20% Knockout Serum Replacement, 2 mM GlutaMAX, 0.1 mM nonessential amino acids (NEAA), 55 μM β-mercaptoethanol, 4 ng/ml FGF-2 and 1% penicillin/streptomycin) for 3 days. Then the EBs were transferred to Matrigel-coated plates in hMSC differentiation medium (MEMα medium (GIBCO) supplemented with 10% Fetal Bovine Serum (FBS), 0.1 mM NEAA, 10 ng/mL FGF-2, 5 ng/mL TGF-β, and 1% penicillin/streptomycin) for approximately 10 days. The cells were then maintained in hMSC culture medium (MEMα medium (GIBCO) supplemented with 10% FBS, 0.1 mM NEAA, 1 ng/mL FGF-2, and 1% penicillin/streptomycin) until 90% confluence and subjected to purification with antibodies corresponding to hMSC-specific markers (CD73, CD90, and CD105) by Fluorescence-Activated Cell Sorting (FACS). The triple-positive cells were cultured for future experiments.

Primary hMSCs (hPMSCs) were isolated from the gingiva tissue of a female individual aged 16. Briefly, the tissue was cut into small pieces and digested in TrypLE™ Express Enzyme plus dispase IV for 30 min at 37°C. Cells were collected and cultured in hMSC culture medium with a gelatin-coated plate.

Cell culture

Antler stem cells (ASCs) (Wang et al., 2019), hMSCs and hPMSCs were cultured in hMSC culture medium. To observe the senescent phenotypes, and the rejuvenation effects of exosomes, hMSCs and hPMSCs in late passage (P12) were used in this study.

Exosome isolation and identification

To collect ASC-derived exosomes, ASCs were cultured in the hMSC culture medium containing 10% FBS in which the exosomes have been removed by ultracentrifugation at 100,000 g for 16 h. Then, the conditioned medium from ASCs at passage 20 was harvested after 48 h and centrifuged for 15 min at 1,500 g to remove cell pellets. Supernatants were then filtered through a 0.22 μm filter. Exosomes were pelleted by ultracentrifugation at 100,000 g for 2 h. The exosome pellets were then washed with 1

mL PBS, pelleted again by ultracentrifugation and resuspended in PBS. Exosomes were measured by negative-staining transmission electron microscopy (TEM) and nanoparticle tracking analysis (NTA).

Immunofluorescence staining

To detect LAP2 and HP1α in hMSCs and hPMSCs, cells were first cultured on a coverslip (Thermo Fisher Scientific). After fixation by 4% PFA, the cells were blocked in 5% BSA-PBS for 30 min at room temperature, and then permeabilized in 0.4% Triton X-100 for 10 min. Primary antibodies diluted in PBS with 1% BSA were added to incubate cells at 4°C overnight. Cells were then washed with PBS and incubated with fluorescent-labeled secondary antibodies and Hoechst 33258 at room temperature for 1 h. The images were captured by a laser scanning confocal microscope and quantified using ImageJ. Antibodies used in this study are listed in Table S4.

SA-β-gal staining

SA-β-gal staining of cultured cells was performed as previously described (Bi et al., 2020). Briefly, cells were firstly fixed with 2% formaldehyde and 0.2% glutaraldehyde for 5 min at room temperature, and then stained with 1 mg/mL X-gal in staining buffer overnight at 37°C. The images were captured and the percentage of SA-β-gal-positive cells was quantified by ImageJ.

Clonal expansion assay

Clonal expansion assay was performed as previously described with some modifications (Cheng et al., 2019). Briefly, cells were seeded at a density of 3×10^3 cells per well in gelatin-coated 12-well plates. Vehicle or ASC-derived exosomes (1×10^8 in 10 μL PBS) were added into the culture medium. The relative crystal violet-stained area was measured by ImageJ.

Cell cycle analysis

Vehicle or exosome-treated cells were collected and fixed in pre-cooled 70% ethanol overnight at -20°C. Cells were then washed once with PBS and stained in 0.02 mg/mL propidium iodide solution containing 0.1% Triton X-100 and 0.2 mg/mL RNase A in PBS at 37°C for 30 min. The samples were then tested with an LSRFortessa cell analyzer (BD) and analyzed using the FlowJo software.

Western blotting

Total protein was isolated from vehicle or exosome-treated cells and quantified using a BCA kit. 20 μg of total protein was separated via SDS-PAGE electrophoresis and then transferred to PVDF membrane. After blocking with 5% non-fat milk, the membrane was first incubated with primary antibodies at 4℃ overnight and then with HRP-conjugated secondary antibodies at room temperature for 1 h. The band visualization was performed by a ChemiDoc XRS system and the data were analyzed by ImageJ. Antibodies used in this study are listed in Table S4.

Animal experiments

The animal experiments were approved by the Experimental Animal Ethics Committee of the Institute of Zoology, Chinese Academy of Sciences, and all experimental mice were treated reasonably. For the OA model, anterior cruciate ligament transection (ACLT) surgery was performed in 8 weeks aged male C57BL/6J mice under anesthesia according to our previous study (Ren et al., 2019). For exosome injection, 1×10^8 ASC-derived exosomes in 10 μL PBS were locally injected into articular cavity after 1 week of ACLT surgery with a frequency of once a week for 8 times. 9 weeks after surgery, grip strength test was performed to evaluate the joint functions, and micro-CT (PerkinElmer) was used to assess the bone mineral density. Afterwards, mice were euthanized and the hindlimb joints were collected for RNA analyses and histological assessment.

Grip strength test

The grip strength was tested according to a previous study with some modifications (Montilla-Garcia et al., 2017). The mice were gently held by the tail and allowed to grasp the gauge with all four paws. They were gently pulled backwards by the tail until they lost their grip. The peak tension in each test was recorded automatically by the device. Each mouse was tested 10 times with 1 min rest between individual tests. The final grip strength was presented as the average of the peak tensions from ten measurements, excluding the maximum and minimum values.

Micro-CT imaging

Bone architecture of whole joint was scanned using X-ray micro-computed tomography (micro-CT) (Yu et al., 2013; Lv et al., 2014) with the following specifications: voxel size 18 μm, voltage 90 kV, current 160 μA, exposure time 1 s. After scanning, the proximal part of the tibias and the distal part of the femurs were selected as a region of interest for three-dimensional reconstruction and bone mineral density analysis using Caliper micro-CT Analyze software (version 10.0).

Histology examination and immunohistochemistry

After being fixed in 4% PFA and decalcified in 5% methanoic acid for two weeks, mouse joints were embedded in paraffin for section preparation (10 μm thickness). For histology analysis, the sections were deparaffinized, rehydrated, stained in Fast Green FCF (0.02%) and safranin O (0.1%) and quantified according to the Osteoarthritis Research Society International (OARSI) scoring system (Wu et al., 2019). For immunohistochemistry, the deparaffinized and rehydrated sections were subjected to antigen retrieval and endogenous peroxidase blockage. Then, sections were blocked in 5% BSA in PBS for 30 min at room temperature, followed by primary antibody incubation overnight at 4℃. The next day, cells were visualized according to the instructions of Histostain SP Kit (ZSGB-BIO) and DAB substrate kit (ZSGB-BIO). The images were captured and then analyzed using ImageJ. Antibodies used in this study are listed in Table S4.

RNA analyses

Total RNA was extracted using TRIzol Reagent from cultured human cells or mouse joints. cDNA was generated with the GoScript Reverse Transcription System (Promega) and used as a template for RT-qPCR detection. qPCR was performed using SYBR qPCR Mix (TOYOBO) in a CFX-384 Real-Time PCR system (Bio-Rad). The h*GAPDH or* m*β-actin* transcript was detected as the internal control. Primers used in this study were listed in Table S5. For cultured cells, two biological replicates were examined in each group. For mouse joints, the RNA samples from the same group (n = 15 mice) were mixed equally in mass, and RNA-seq was performed with three technical replicates. Sequencing libraries were constructed using a Next Ultra RNA Library Prep Kit for Illumina (NEB) following the manufacturer's protocol. The libraries were then sequenced on Illumina HiSeq X-Ten platforms with paired-end sequencing at 150-bp

read length. Quality control and sequencing were performed by Novogene Bioinformatics Technology.

Quantitative proteomics analysis

Extracted proteins from ASC exosomes were separated on a 10% SDS-PAGE gel and stained with Coomassie brilliant blue. Gel slices containing protein bands of interest underwent decolorization and hydrolyzation overnight. The peptides were extracted by different concentrations of acetonitrile. The obtained peptide mixture was analyzed by NanoLC-Q Exactive, and then proteins were identified by using the sequence HT search engine of thermo proteome discoverer (1.4.0.288) in the UniProt organization Cervus combined with contaminants database. A few bovine serum proteins were identified and removed from the next step analysis. Gene Ontology enrichment analysis was conducted by Metascape (http://metascape.org/gp/). Identified proteins are listed in Table S1.

RNA-seq data processing

The RNA-seq raw data was trimmed using Trim Galore (version 0.5.0) (https://github.com/FelixKrueger/TrimGalore) to remove low-quality reads and reads with adapters. The cleaned data was mapped with the mouse mm10 genome or the human reference genome (hg19) using HISAT software (version 2.0.4) (Kim et al., 2015). Then, the mapped data was counted using HTSeq (version 0.11.0) (Anders et al., 2015). Differentially expressed genes (DEGs) were calculated using the R package DESeq2 (version 1.26.0) (Love et al., 2014) with the Benjamini-Hochberg adjusted *P*-value cutoff set as less than 0.01 in mouse dataset and less than 0.05 in the human dataset, while absolute fold-change cutoff set as more than $\log_2$ (1.5) for both datasets. Enrichment analysis was performed using Metascape (http://metascape.org) (Zhou et al., 2019). The DEGs are listed in Table S2. The upstream regulators of DEGs were analyzed using the "Upstream Regulator Analysis" tool of Ingenuity Pathway Analysis (IPA, http://www.ingenuity.com) software. The plots were generated using the R package ggplot2 (version 3.3.3). The upstream regulators are listed in Table S3.

Statistical analysis

Data were expressed as mean ± SEMs. Two-tailed Student's t test was used for comparing the difference between groups. Multi-group comparisons were performed by one-way ANOVA followed by Dunnett's test. GraphPad Prism 8.0 was used for statistical analysis. *P*-value less than 0.05 was considered as statistically significant.

Data Availability

RNA-seq data have been deposited in the NCBI Gene Expression Omnibus (GEO) under the accession number GSE172430, and also in the Aging Atlas database (https://bigd.big.ac.cn/aging/index).

Supplementary Figure 1. Quantitative proteomics analysis of ASC-derived exosomes and their effects on human MSCs and mouse OA.

(A) Schematic diagram of the LC-MS/MS strategy for identifying proteins from ASC-derived exosomes.

(B) Gene ontology (GO) analysis of the identified proteins from ASC-derived exosomes. The top three proteins enriched in each pathway were shown in red.

(C) The top 10 proteins identified in ASC-derived exosomes based on their score value were listed in the table.

(D) Principal component analysis (PCA) showing the correlation between RNA-seq replicates of hPMSCs treated by Veh or Exo.

(E) Heatmap showing the relative expression levels of indicated DEGs in Veh or Exo-treated hPMSCs.

(F) Principal component analysis (PCA) showing the correlation among replicates of RNA-seq analysis of the mouse joints.

(G) Venn diagrams showing the overlap of DEGs in Exo-treated hPMSCs and rescued DEGs in Exo-treated OA mouse joints. Specific genes corresponding to indicated heatmaps were listed to the right.

Supplementary Table Legends

Table S1. Proteins from ASC-derived exosomes identified by quantitative proteomics analysis

Table S2. DEGs identified by RNA-seq analysis of hPMSCs and mouse joints

Table S3. The upstream regulators predicted by Ingenuity Pathway Analysis

Table S4. Antibodies used in this study

Table S5. Primers used for RT-qPCR

Reference

Anders, S., Pyl, P.T., and Huber, W. (2015). HTSeq--a Python framework to work with high-throughput sequencing data. Bioinformatics 31, 166-169.

Bi, S., Liu, Z., Wu, Z., Wang, Z., Liu, X., Wang, S., Ren, J., Yao, Y., Zhang, W., Song, M., et al. (2020). SIRT7 antagonizes human stem cell aging as a heterochromatin stabilizer. Protein Cell 11, 483-504.

Cheng, F., Wang, S., Song, M., Liu, Z., Liu, P., Wang, L., Wang, Y., Zhao, Q., Yan, K., Chan, P., et al. (2019). DJ-1 is dispensable for human stem cell homeostasis. Protein Cell 10, 846-853.

Kim, D., Langmead, B., and Salzberg, S.L. (2015). HISAT: a fast spliced aligner with low memory requirements. Nat Methods 12, 357-360.

Liang, C., Liu, Z., Song, M., Li, W., Wu, Z., Wang, Z., Wang, Q., Wang, S., Yan, K., Sun, L., et al. (2021). Stabilization of heterochromatin by CLOCK promotes stem cell rejuvenation and cartilage regeneration. Cell Res 31, 187-205.

Love, M.I., Huber, W., and Anders, S. (2014). Moderated estimation of fold change and dispersion for RNA-seq data with DESeq2. Genome Biol 15, 550.

Lv, Y., Xia, J.Y., Chen, J.Y., Zhao, H., Yan, H.C., Yang, H.S., Li, Q., Fan, Y.X., Guo, K.J., and Chen, X.Y. (2014). Effects of pamidronate disodium on the loss of osteoarthritic subchondral bone and the expression of cartilaginous and subchondral osteoprotegerin and RANKL in rabbits. BMC Musculoskelet Disord 15, 370.

Montilla-Garcia, A., Tejada, M.A., Perazzoli, G., Entrena, J.M., Portillo-Salido, E., Fernandez-Segura, E., Canizares, F.J., and Cobos, E.J. (2017). Grip strength in mice with joint inflammation: A rheumatology function test sensitive to pain and analgesia. Neuropharmacology 125, 231-242.

Ren, X., Hu, B., Song, M., Ding, Z., Dang, Y., Liu, Z., Zhang, W., Ji, Q., Ren, R., Ding, J., et al. (2019). Maintenance of Nucleolar Homeostasis by CBX4 Alleviates Senescence and Osteoarthritis. Cell Rep 26, 3643-3656 e3647.

Wang, D., Berg, D., Ba, H., Sun, H., Wang, Z., and Li, C. (2019). Deer antler stem cells are a novel type of cells that sustain full regeneration of a mammalian organ-deer antler. Cell Death Dis 10, 443.

Wu, J., Kuang, L., Chen, C., Yang, J., Zeng, W.N., Li, T., Chen, H., Huang, S., Fu, Z., Li, J., et al. (2019). miR-100-5p-abundant exosomes derived from infrapatellar fat pad MSCs protect articular cartilage and ameliorate gait abnormalities via inhibition of mTOR in osteoarthritis. Biomaterials 206, 87-100.

Yu, D., Liu, F., Liu, M., Zhao, X., Wang, X., Li, Y., Mao, Y., and Zhu, Z. (2013). The

inhibition of subchondral bone lesions significantly reversed the weight-bearing deficit and the overexpression of CGRP in DRG neurons, GFAP and Iba-1 in the spinal dorsal horn in the monosodium iodoacetate induced model of osteoarthritis pain. PLoS One 8, e77824.

Zhou, Y., Zhou, B., Pache, L., Chang, M., Khodabakhshi, A.H., Tanaseichuk, O., Benner, C., and Chanda, S.K. (2019). Metascape provides a biologist-oriented resource for the analysis of systems-level datasets. Nat Commun 10, 1523.

Supplementary Figure 1

A

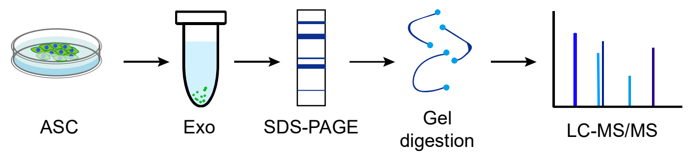

B

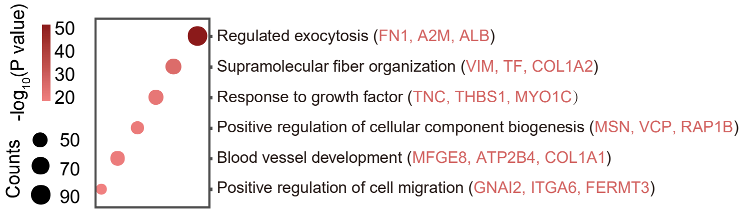

C

Protein name	Abbreviation	Function	Score	Coverage (%)	Unique peptide
Fibronectin	FN	Enhancement of migration and invasion<br>Increase in survival and proliferation	619.04	52.6	106
Milk fat globule EGF factor 8	MFGE8	Angiogenesis<br>Apoptotic cell clearance	279.84479	68.35	41
Clathrin heavy chain	CLTC	Intracellular protein transport	273.24401	47.45	58
PDZ domain-containing protein	PDZD	Interaction with ligands<br>Protein interaction	238.38495	45.45	92
Vitellogenin domain-containing protein	APOB	Post-embryonic development	216.67797	20.16	70
Alpha-2-Macroglobulin	A2M	Inhibit inflammatory cascade	205.19367	28.9	2
Albumin	ALB	Negative regulation of apoptotic process<br>Negative regulation of programmed cell death	169.05178	36.36	4
Tenascin C	TNC	Positive regulation of cell proliferation	151.02396	34.2	35
Major vault protein	MVP	Promote cell proliferation and survival<br>Inflammatory suppression	141.49855	55.09	39
Moesin	MSN	Positive regulation of gene expression	101.89907	52.05	29

D

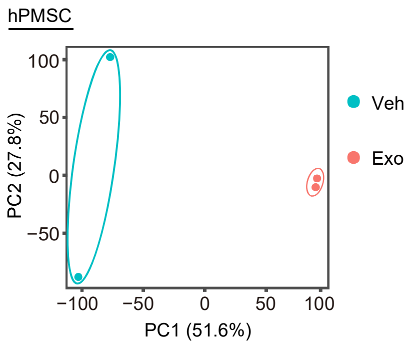

E

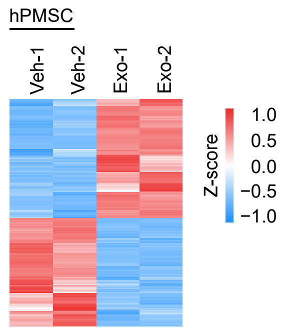

F

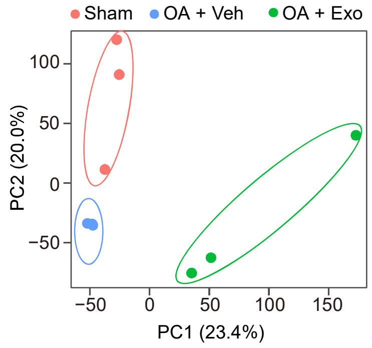

G

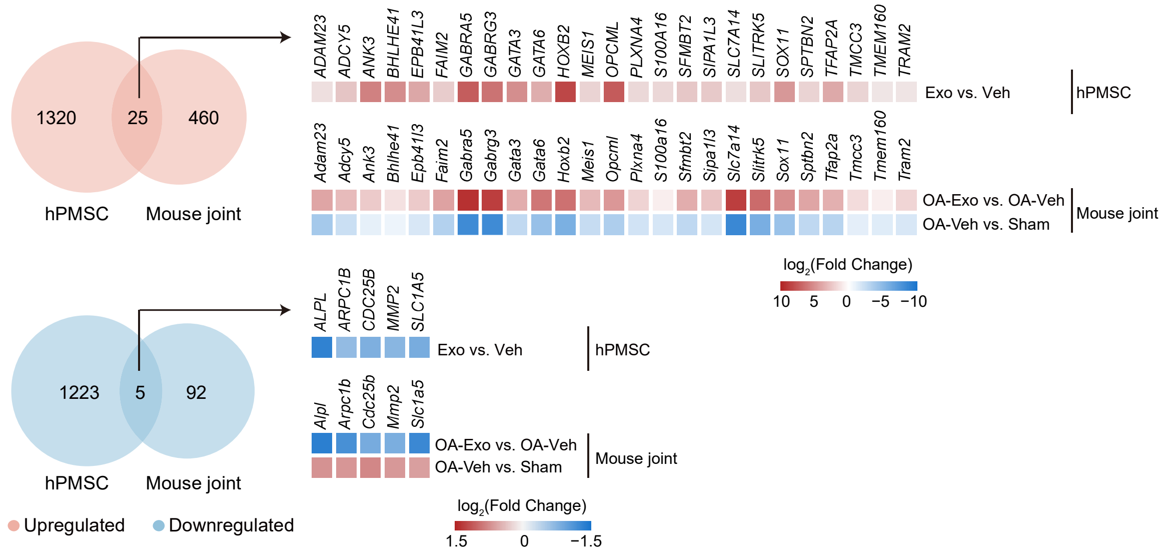

Supplement: Supplementary file 1 — Supplementary material 1 (PDF 556 kb) [file 13238_2021_860_MOESM1_ESM.pdf]
